# Identification of the *FtsH* gene family in chrysanthemums and functional analysis of *CmFtsH-15* under cadmium stress

**DOI:** 10.3389/fpls.2026.1768126

**Published:** 2026-03-13

**Authors:** Liuliu Wu, Lifan Cao, Zongli Chu, Liping Dong, Junyan Sun, Shumei Li, Yanlin Yang, Shuhan Liu, Shengli Tong, Mingming Tang, Halyna Zhatova, Li Meng

**Affiliations:** 1 College of Agriculture, Xinyang Agriculture and Forestry University, Xinyang, China; 2 Department of Biology, Sumy National Agrarian University, Sumy, Ukraine; 3 College of Agriculture, Henan Institute of Science and Technology, Xinxiang, China

**Keywords:** Cd stress, *Chrysanthemum morifolium*, CmFtsH-15, *FtsH* gene family, protein-protein interaction

## Abstract

The *FtsH* gene family encodes ATP-dependent zinc metalloproteases essential for protein quality control, organelle homeostasis, and stress response in plants. Nevertheless, research on the *FtsH* gene family in *Chrysanthemum morifolium* is limited. This study identified 32 *CmFtsH* genes through bioinformatics approaches and systematically analyzed their family members. Phylogenetic analysis clarified their evolutionary relationships, while chromosomal localization, sequence alignment, and promoter *cis*-element prediction were utilized to analyze gene characteristics. Tissue-specific expression profiling identified key genes, and overexpression experiments confirmed the cadmium (Cd) tolerance of the candidate gene *CmFtsH-15*. The analysis indicated a close evolutionary relationship with *Asteraceae* plants such as lettuce and sunflower, demonstrating lineage-specific differentiation. The 32 *CmFtsH* genes are unevenly distributed across 16 chromosomes, exhibiting significant differences in sequence length and motif composition. Promoter regions are abundant in stress and hormone response elements, indicating potential involvement in abiotic stress adaptation. *CmFtsH-15* is significantly expressed in leaves, and its overexpression alleviates oxidative damage by reducing Cd accumulation, enhancing antioxidant activity, and decreasing malondialdehyde content, thereby enhancing Cd tolerance in transgenic lines. Furthermore, CmFtsH-15 interacts with the heat shock protein CmHSP70, suggesting a synergistic regulation of stress response. This study systematically explored the *FtsH* gene family in *Chrysanthemum*, highlighting the protective role of *CmFtsH-15* under Cd stress, thus providing a promising candidate for developing Cd-resistant germplasm resources.

## 1 Introduction

FtsH proteins are ATP-dependent zinc metalloproteinases that are evolutionarily conserved (Wu et al., 2021). The FtsH protease was initially identified in *Escherichia coli* as a metalloprotease (Wagner et al., 2012). FtsH family members form functional homo- or heterohexamers (Yi et al., 2022). Each FtsH protease consists of an N-terminal transmembrane segment and a C-terminal region containing the AAA-ATPase structural domain, which is classified within the M41 peptidase family of protease domains (Wu et al., 2021). These proteins play a critical role in degrading misfolded or damaged proteins, thus maintaining intracellular homeostasis and organelle integrity. FtsH proteins are highly conserved across species, from bacteria to higher plants, underscoring their fundamental role in cellular processes (Wagner et al., 2012). The AAA+ structural domain, characteristic of FtsH proteins, facilitates ATP hydrolysis, which provides the energy required for protein unfolding and degradation. The zinc-binding domain is essential for the proteolytic activity of FtsH proteins. These conserved domains allow FtsH proteins to recognize and degrade a diverse array of target proteins, supporting their multifunctional roles in cellular homeostasis and stress responses (Langklotz et al., 2012).

FtsH proteins perform numerous essential functions in plants. In higher plants, FtsH proteins are implicated in chloroplast development, photosynthesis, and responses to abiotic stress (Wang et al., 2025a). The functional diversity of FtsH proteins enables their localization in multiple cellular compartments, including chloroplasts, mitochondria, and cytoplasm (Mawla et al., 2024). During photosynthesis, the core D1 protein of Photosystem II (PSII) is particularly susceptible to photo-oxidative damage. The FtsH protease degrades damaged D1 protein, thereby aiding the repair of the PSII complex. This mechanism maintains stable photosynthetic efficiency under intense light, optimizing plant adaptation to varying light environments (Wang et al., 2025b; Kato and Sakamoto, 2018). *FtsH* genes are pivotal to normal chloroplast development. For instance, mutations within the *FtsH2* gene in *Arabidopsis thaliana* lead to irregular chloroplast development, evidenced by the emergence of green spots on foliage (Zhang et al., 2010). Similarly, the knockdown of the FtsH gene in rice results in plant albinism, emphasizing the essential role of this gene in chloroplast synthesis and development (Wu et al., 2021). Gene ontology (GO) annotations show the involvement of the *FtsH* gene in plant embryo and leaf development (Pu et al., 2022), highlighting the significant role FtsH proteins play throughout plant growth and maturation. Additionally, FtsH proteins localize to the mitochondrial membrane, contributing to the homeostatic regulation of mitochondrial proteins. This localization suggests FtsH proteins significantly influence energy metabolism and respiratory processes within plant cells. High-temperature stress negatively impacts photosynthesis and protein stability in plants. FtsH proteins are strongly correlated with plant resilience to stress factors, including drought, UV radiation, and high salinity. By regulating proteostasis in chloroplasts and other organelles, FtsH proteins enhance plant survivability under adverse conditions. Specifically, their role in D1 protein turnover under high-temperature conditions mitigates the inhibitory effects of heat stress on photosynthesis (Xiao et al., 2021; Hajibarat and Saidi, 2023). In addition to their protease activity, FtsH proteins function as molecular chaperones, assisting in proper protein folding. This activity sustains the structural integrity and functional capacity of intracellular proteins, thereby supporting normal physiological processes within plant cells (Li et al., 2025).

Chrysanthemum is an economically and horticulturally important species, extensively cultivated for ornamental purposes and traditional medicinal applications. Deciphering the genetic mechanisms underlying chrysanthemum tolerance to stress is vital for breeding varieties that can endure adverse environmental conditions, including heavy metal contamination (Su et al., 2024). Cadmium (Cd), among heavy metals, is particularly toxic and poses a significant threat to plant growth and human health (Li et al., 2023). Previous research has demonstrated the pivotal role of FtsH proteins in detoxifying heavy metals. These proteins alleviate heavy metal toxicity by degrading damaged photosynthetic and organelle proteins, thereby assisting plants in maintaining physiological functions (Huang et al., 2024). The function of the *TaFtsH-1* gene was investigated using barley streak mosaic virus-induced gene silencing (*BSMV*-VIGS), revealing that *TaFtsH-1* gene silencing increased wheat tolerance to Cd toxicity (Huang et al., 2024). Further, the expression level of the *TaFtsH-1* gene was significantly upregulated in plants exposed to Cd stress, presumably to bolster FtsH protein activity against Cd-induced oxidative stress and protein damage. *FtsH* genes functioned to degrade proteins damaged by reactive oxygen species accumulated during Cd stress, thereby minimizing oxidative damage at the cellular level (Tang et al., 2016). Identifying and characterizing the *FtsH* gene family in chrysanthemums could yield valuable insights into Cd tolerance mechanisms and facilitate the development of chrysanthemum varieties with enhanced resistance to environmental stressors.

In recent years, genome-wide characterization and functional analyses have confirmed the essential role of FtsH proteins in plant responses to various stresses. For instance, water deficit stress combined with abscisic acid (ABA) treatment significantly upregulated the transcription of *ZmFtsH2B* in maize leaves (Yue et al., 2010). The *CaFtsH1* and *CaFtsH8* genes play vital roles in chloroplast development and functionality in chili peppers. Silencing each of *CaFtsH1* or *CaFtsH8* disrupted leaf development in peppers, emphasizing their critical roles in chloroplast development and photosynthesis regulation (Xu et al., 2023). Under light stress conditions, overexpression of *GmFtsH25* promoted vesicle stacking in chloroplasts, enhanced photosynthetic efficiency, and elevated starch accumulation (Wang et al., 2023). Similarly, overexpression of alfalfa *MsFtsH8* in Arabidopsis demonstrated its regulatory role in enhancing salt tolerance (Li et al., 2024). In this study, we performed a comprehensive analysis of the *FtsH* gene family in chrysanthemums, identifying 32 *CmFtsH* genes and characterizing their phylogenetic relationships, gene structures, chromosomal locations, and *cis*-acting elements. Additionally, we analyzed the expression patterns of these genes across various tissues. A functional analysis of *CmFtsH-15* was performed, confirming its essential role in mitigating Cd stress in chrysanthemums. To analyze the potential mechanisms through which the *CmFtsH* genes participate in the Cd stress response, this study employed protein interaction complementation assays to identify proteins that interact with CmFtsH proteins. These proteins may collaborate with the *CmFtsH* gene complex under Cd stress, indirectly enhancing plant Cd tolerance by maintaining protein homeostasis and organelle integrity, thus contributing to a more comprehensive understanding of its molecular mechanisms. The insights gained from this research may establish a basis for enhancing resistance in chrysanthemums and related species.

## 2 Materials and methods

### 2.1 Database Search of the *FtsH genes*

The *FtsH* gene family data used in this study were acquired from chrysanthemum varieties provided by the Henan Institute of Science and Technology. Complete genome sequences of *Arabidopsis thaliana*, *Chrysanthemum morifolium*, *Lactuca sativa*, and *Helianthus annuus* were obtained from the respective databases: TAIR (https://www.arabidopsis.org/) (Huala et al., 2001), Chrysanthemum Genome Database (http://www.amwayabrc.com/zh-cn/index.html) (Ye et al., 2024), Lettuce Database (https://www.db.cngb.org/lettuce/) (Guo et al., 2023), and Sunflower Database (http://www.helianthome.org) (Bercovich et al., 2022). Additionally, RNA-Seq data were procured from the Chrysanthemum Genome Database for various tissues, including disc floret petals, disc floret pistils, disc floret stamens, ray floret petals, ray floret pistils, roots, shoots, stems, and leaves.

### 2.2 Identification of *FtsH* genes

The HMM profile for the FtsH domain (PF00004) was accessed from the Pfam protein family database (http://pfam.xfam.org/) (Finn et al., 2014). The HMMER3 hmmsearch tool was employed to identify FtsH protein sequences within the genomes of *Chrysanthemum morifolium*, *Lactuca sativa*, and *Helianthus annuus*, using an E-value threshold of less than 0.01. Subsequently, the sequences identified were further validated using the Pfam and SMART databases (http://smart.embl-heidelberg.de/) (Schultz et al., 2000) to verify the presence of FtsH structural domains. A comprehensive list of FtsH protein sequences was compiled by removing redundant sequences and confirming the incorporation of structural domains in all selected entries.

### 2.3 Physicochemical characterization and phylogenetic analysis

The ProtParam tool (Gasteiger et al., 2005), available at https://web.expasy.org/protparam/, was employed to calculate the molecular weight, theoretical isoelectric point (pI), and additional physicochemical properties of the CmFtsH proteins. The amino acid sequences of the identified *101* FtsH proteins were used to construct a phylogenetic tree. The ClustalW algorithm was employed for multiple sequence alignments, and the phylogenetic tree was generated using the neighbor-joining method with 1,000 bootstrap replicates using MEGA11 software (Tamura et al., 2021). Evolview 3.0 software (Zhang et al., 2012) was employed for visualization and enhancement of the phylogenetic tree.

### 2.4 Chromosomal localization and gene structure analysis

The chromosomal locations and gene structure data of members of the *CmFtsH* gene family were obtained from the downloaded chrysanthemum genome annotation file (GFF3) through the chrysanthemum Genome Database. Analysis of conserved motifs within the *CmFtsH* gene family was conducted using the MEME suite (https://meme-suite.org/meme/tools/meme) (Bailey et al., 2015), setting the maximum number of motifs to 10, while maintaining default settings for all other parameters. Visualization of chromosomal localization, protein conserved motifs, and gene structures was achieved using TBtools software (Chen et al., 2023).

### 2.5 Prediction of *Cis*-acting elements

Promoter sequences located 2000 base pairs upstream of the *CmFtsH* genes initiation codon were extracted from the chrysanthemum genome annotation file. *Cis*-regulatory elements in these promoter sequences were identified using the PlantCARE database (Lescot et al., 2002). The results were visualized using TBtools software.

### 2.6 Collinearity analysis

The whole genome sequences and annotation files in GFF3 format were obtained from the chrysanthemum Genome Database and analyzed for collinearity with the Multiple Collinearity Scan Toolkit (MCScanX) (Wang et al., 2012), and the results were subsequently visualized with TBtools software.

### 2.7 Ontology annotation analysis

The Gene Ontology (GO) annotation for *CmFtsH* genes was performed using the Database for Annotation, Visualization, and Integrated Discovery (DAVID) (Sherman et al., 2022). After applying a -log_10_ transformation to the p-values, the results of the GO annotation were visualized using the Chiplot online platform (https://www.chiplot.online/).

### 2.8 Expression pattern analysis

Using the comprehensive *CmFtsH* genes list obtained from this study as a template, the first ten unstable junctions in the bipartite RNA-Seq data from various chrysanthemum tissues were removed. Subsequently, the Kallisto software was used to calculate the log_2_ (TPM + 1) values, and a transcriptome expression heat map was created using TBtools software.

### 2.9 Transgenic chrysanthemum preparation and Cd stress tolerance evaluation

The complete cDNA sequence of *CmFtsH-15* was amplified using PCR with gene-specific primers incorporating *Xba* I and *Sac* I restriction sites (**Supplementary Table 1**). The obtained sequence was subsequently inserted into the pBI121 vector, fused with the Cauliflower mosaic virus 35S (CaMV35S) promoter. Transgenic plants overexpressing *CmFtsH-15* were generated through *Agrobacterium*-mediated transformation (Bi et al., 2021).

To investigate the role of *CmFtsH-15* under Cd stress, wild-type (WT) plants and transgenic plants overexpressing *CmFtsH-15* (homozygous T2 generation) were cultivated in Hoagland nutrient solution (Dai et al., 2012) and subjected to 2 mM Cd treatment (Alzahrani et al., 2018). After 30 days, plant growth was recorded, and relevant indices such as plant height and dry weight were measured. To study the expression pattern of transgenic plants after Cd stress, the *CmEF1α* was used as an internal reference gene (**Supplementary Table 1**) (Wang et al., 2015). Total RNA was extracted from chrysanthemum samples using the TRIZOL reagent (TIANGEN, Beijing, China). Complementary DNA (cDNA) was synthesized from the extracted RNA through reverse transcription using the PrimeScript RT reagent kit (Takara, Japan). Subsequently, quantitative real-time PCR (qRT-PCR) was conducted using SYBR Premix Ex Taq II (TaKaRa, Japan) on an ABI 7500 Real-Time PCR System (Thermo Fisher Scientific, USA) for gene expression analysis.

Additionally, the activity of antioxidant enzymes including catalase (CAT), peroxidase (POD) and superoxide dismutase (SOD) (Nanjing Jiancheng, A007-2-1, A084-1, A001-3-2, China), and malondialdehyde (MDA) (Nanjing Jiancheng, A003-4, China) were analyzed using assay kits to evaluate the response to oxidative stress.

Transmission electron microscopy (TEM) was performed according to the protocol (Kong et al., 2013), with minor modifications. Ultrathin sections of root tips were analyzed using a Hitachi HT7700 TEM set to operate at 80 kV. A minimum of three biological replicates were analyzed for each treatment, and representative images were selected for presentation.

To determine Cd concentration, tissue samples from transgenic chrysanthemums were washed with deionized water and then dried at 80 °C for 72 hours. Each sample, weighing 0.5 g, was precisely measured, and 5 mL of HNO_3_ and 2 mL of H_2_O_2_ were added. The samples were subjected to microwave-assisted digestion using a stepwise temperature program of 160 °C, 110 °C, and 160 °C, each step lasting 30 minutes. After digestion, the samples were analyzed via inductively coupled plasma mass spectrometry (ICP-MS, model Puxi TAS-986, China) (Zhang et al., 2018) to measure Cd levels.

### 2.10 Protein-protein interaction analysis

The interaction between CmFtsH-15 and CmHSP70 (heat shock protein) was predicted using the STRING database (https://string-db.org/). This interaction was subsequently validated through yeast two-hybrid (Y2H) assays, luciferase (Luc) assays, and bimolecular fluorescence complementation (BiFC) assays.

The Y2H assay was conducted as previously described (Feng et al., 2021) to validate the interaction between CmFtsH-15 and CmHSP70. The complete coding sequence (CDS) of *CmHSP70* was cloned into the pGADT7 vector and co-transfected with the *CmFtsH-15*-pGBKT7 recombinant plasmid into Y2HGold competent cells. The transformed cells were subsequently plated onto SD/-Trp/-Leu and SD/-Trp/-Leu/-His/-Ade deficient media to observe growth conditions.

For the BiFC analysis, the CDSs of *CmFtsH-15* and *CmHSP70* were cloned into the pXY106-nYFP and pXY104-cYFP vectors, respectively. Subsequently, the nYFP-CmFtsH-15 + pXY104 and pXY106 + CmHSP70-cYFP fusion expression vectors were transformed into *Agrobacterium tumefaciens*. The bacterial solutions were mixed in a 1:1 ratio and injected into tobacco (*Nicotiana benthamiana*). After three days of cultivation, lower epidermal cells from tobacco leaves were collected, and YFP fluorescence was observed utilizing a laser confocal microscope (Olympus FV1200, Japan).

The open reading frames (ORFs) of *CmFtsH-15* and *CmHSP70* were inserted into the pCAMBIA-nLuc and pCAMBIA-cLuc vectors, respectively, thereby constructing Luc expression vectors. The resultant recombinant plasmids were co-transformed into tobacco leaves, which were subsequently incubated for 48 hours. The fluorescence intensity was detected using a Night Owl LB985 fluorescence detector (Berthold Technologies, Germany).

### 2.11 Statistical analysis

All experiments were performed using three biological replicates. Statistical analysis was conducted using a one-way ANOVA. Tukey’s test was employed to identify significant differences, and the results were presented using GraphPad Prism 8.0 software.

## 3 Results

### 3.1 Physicochemical characterization of proteins from the *CmFtsH* gene family

Comprehending protein families is fundamental for elucidating gene functions and regulatory mechanisms (Zhou et al., 2021). A total of 32 *CmFtsH* gene family members were identified within the chrysanthemum genome through the analysis utilizing HMMER and SMART online tools, using the sequences of the Arabidopsis *FtsH* gene family members as a reference (**Table 1**). These genes are distributed across multiple chromosomes within the chrysanthemum genome, with chromosomes 1 and 12 each containing three family members. For instance, *CmFtsH-4* and *CmFtsH-6* are located on chromosome 1, whereas *CmFtsH-2*, *CmFtsH-12*, and *CmFtsH-14* are located on chromosome 12. The length of amino acids encoded by these genes varies substantially, with the shortest being CmFtsH-17 (173 amino acids) and the longest being CmFtsH-7 (911 amino acids), corresponding to molecular weights ranging from 18.97 kDa (CmFtsH-17) to 99.03 kDa (CmFtsH-7). The pI exhibited considerable variation, ranging from a minimum of 5.58 (CmFtsH-29) for acidic proteins to a maximum of 9.79 (CmFtsH-14) for basic proteins, indicating the presence of both acidic and basic proteins within the family. Such characteristics align with the role of FtsH proteins in regulating organelle function (Yi et al., 2022; Pu et al., 2022).

**Table 1:** Characteristic of *FtsH* gene family in *Chrysanthemum morifolium*.

Gene name	Sequence_ID	Gene_locus	Chr	Number of amino acids	Molecular weight (kDa)	Theoretical pI	Instability index
*CmFtsH-1*	*evm.model.scaffold_1613.199*	*182313981-182314031*	*11*	815	89.92	7.18	37.81
*CmFtsH-2*	*evm.model.scaffold_521.64*	*201961043-201961093*	*12*	815	89.81	7.58	36.74
*CmFtsH-3*	*evm.model.scaffold_1527.132*	*202702395-202702445*	*10*	815	89.90	6.88	37.22
*CmFtsH-4*	*evm.model.scaffold_1256.152*	*183508392-183508517*	*1*	646	71.68	5.83	31.83
*CmFtsH-5*	*evm.model.scaffold_220.244*	*167534007-167534135*	*3*	661	73.45	5.88	30.86
*CmFtsH-6*	*evm.model.scaffold_1256.141*	*183159638-183159727*	*1*	646	71.56	6.04	35.18
*CmFtsH-7*	*evm.model.scaffold_471.17*	*158125231-158125353*	*2*	911	99.03	6.93	42.33
*CmFtsH-8*	*evm.model.scaffold_3344.2*	*181744642-181744692*	*11*	401	44.74	8.85	39.91
*CmFtsH-9*	*evm.model.scaffold_9570.4*	*99055328-99056338*	*25*	611	67.75	9.55	43.28
*CmFtsH-10*	*evm.model.scaffold_1097.8*	*112515508-112516521*	*26*	612	67.67	9.52	43.39
*CmFtsH-11*	*evm.model.scaffold_5841.135*	*111730825-111731838*	*26*	612	67.67	9.52	43.39
*CmFtsH-12*	*evm.model.scaffold_6984.36*	*188932255-188933316*	*12*	353	39.93	9.75	38.59
*CmFtsH-13*	*evm.model.scaffold_155.220*	*212156528-212157589*	*10*	353	39.93	9.75	38.65
*CmFtsH-14*	*evm.model.scaffold_6984.25*	*189014566-189015627*	*12*	353	40.06	9.79	40.16
*CmFtsH-15*	*evm.model.scaffold_6291.23.1*	*266754543-266754884*	*17*	701	75.22	6.06	33.61
*CmFtsH-16*	*evm.model.scaffold_1638.26*	*273619993-273621174*	*18*	692	74.07	5.94	33.56
*CmFtsH-17*	*evm.model.scaffold_362.10*	*150976836-150977357*	*8*	173	18.97	8.56	45.91
*CmFtsH-18*	*evm.model.scaffold_11180.71*	*140694369-140694946*	*7*	670	72.45	5.84	34.23
*CmFtsH-19*	*evm.model.scaffold_225.599*	*149307727-149308304*	*9*	670	72.35	5.94	35.18
*CmFtsH-20*	*evm.model.scaffold_1759.73*	*26287122-26288393*	*7*	698	74.67	5.99	35.09
*CmFtsH-21*	*evm.model.scaffold_6797.119*	*33494745-33496016*	*8*	698	74.64	5.99	35.33
*CmFtsH-22*	*evm.model.scaffold_1759.44*	*36779151-36780500*	*9*	724	77.55	6.18	35.73
*CmFtsH-23*	*evm.model.scaffold_1016.11*	*209165544-209165584*	*13*	782	84.82	6.27	33.16
*CmFtsH-24*	*evm.model.scaffold_1016.35*	*208822606-208823430*	*13*	809	88.18	8.07	31.3
*CmFtsH-25*	*evm.model.scaffold_1436.131*	*185200597-185201421*	*14*	809	88.18	8.07	31.3
*CmFtsH-26*	*evm.model.scaffold_6137.49*	*199705566-199705757*	*7*	590	65.12	8.26	28.45
*CmFtsH-27*	*evm.model.scaffold_629.398*	*205871114-205871308*	*9*	591	65.29	8.26	28.16
*CmFtsH-28*	*evm.model.scaffold_2080.1*	*205892960-205893154*	*9*	591	65.45	8.43	28.36
*CmFtsH-29*	*evm.model.scaffold_87.4*	*101244597-101244623*	*16*	771	86.97	5.58	40.5
*CmFtsH-30*	*evm.model.scaffold_5200.100*	*107771881-107772081*	*17*	829	93.37	5.87	42.59
*CmFtsH-31*	*evm.model.scaffold_asm16_new.2348*	*113285222-113285422*	*18*	829	93.37	5.87	42.59
*CmFtsH-32*	*evm.model.scaffold_1115.61*	*92901437-92901712*	*13*	253	29.25	9.1	52.5

### 3.2 Phylogenetic analysis of the *FtsH* genes

**Figure 1:**
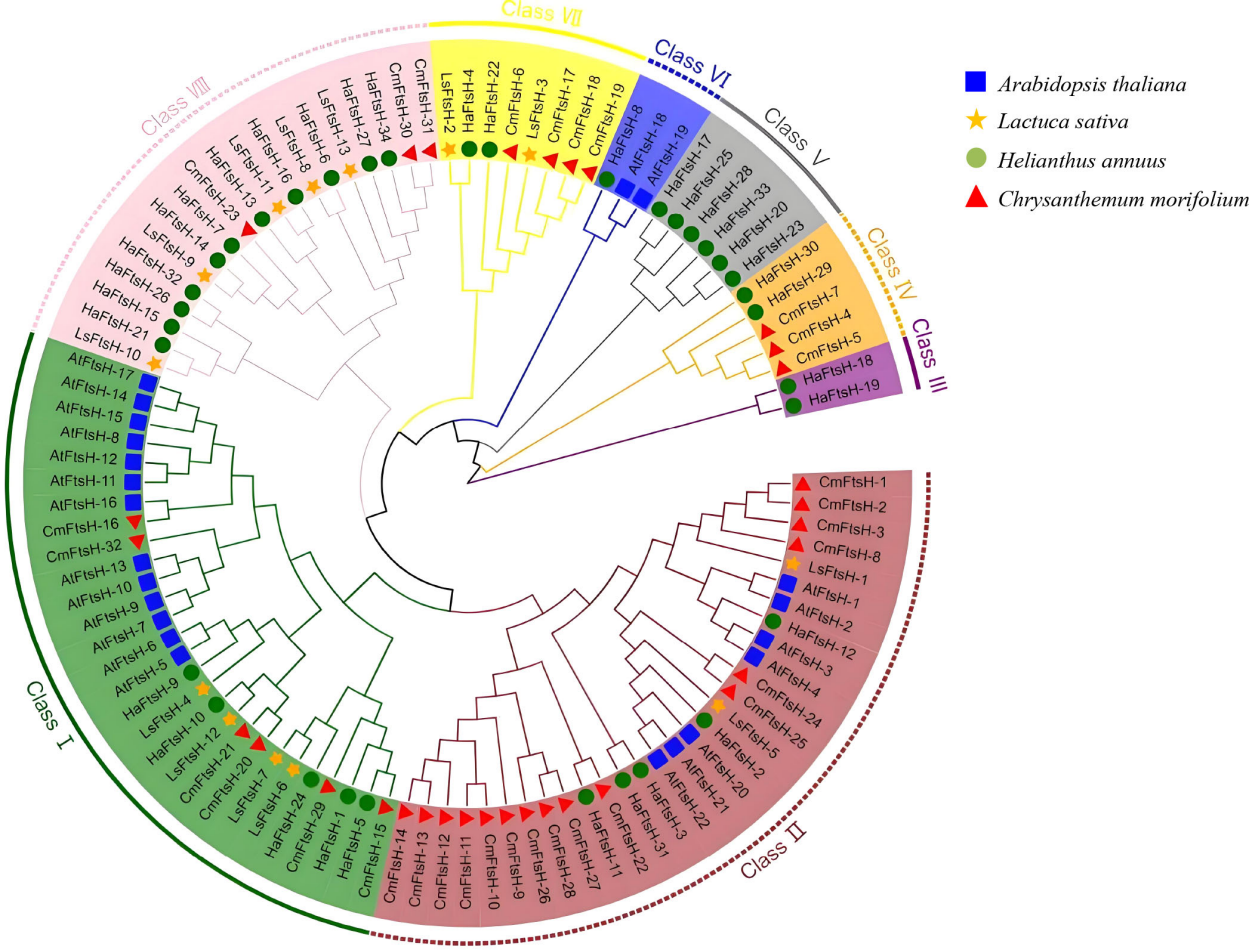
Phylogenetic analysis of *FtsH* genes in chrysanthemum, Arabidopsis, lettuce and sunflower. The different shapes indicated various species. Triangle: Cm, *Chrysanthemum morifolium*; Square: At, *Arabidopsis thaliana*; Star: Ls, *Lactuca sativa*; Roundness: Ha, *Helianthus annuus*. The different background colors indicated the different *FtsH* gene types.

To investigate the evolutionary relationships of *FtsH* genes, phylogenetic trees were constructed for chrysanthemum, Arabidopsis, lettuce, and sunflower using sequences from *Chrysanthemum morifolium* (*CmFtsH*), *Arabidopsis thaliana* (*AtFtsH*), *Lactuca sativa* (*LsFtsH*), and *Helianthus annuus* (*HaFtsH*). As shown in **Figure 1**, *AtFtsH* genes and *CmFtsH* genes are located on different branches, indicating that they have significant evolutionary differences. *FtsH* genes were classified into eight subfamilies (Class I–VIII) based on species relatedness. The majority of *CmFtsH* genes were located in Class II (50%), while most *AtFtsH* genes were distributed across Class I (59.1%) and Class II (31.8%), highlighting their genetic divergence from *Asteraceae* family members. This suggests that *CmFtsH* genes might have followed an evolutionary path similar to their *Asteraceae* relatives. The sunflower *FtsH* genes were classified in Class VIII (32.4%) and Class V (17.6%), whereas lettuce *FtsH* genes were predominantly in Class VIII (38.5%). Within the *CmFtsH* genes, genes in Class II and Class I comprised 50% and 18.7% of the total identified *FtsH* genes in chrysanthemums, respectively (**Figure 1**; **Table 2**). The findings suggest that *FtsH* genes are more conserved within *Asteraceae* species than in non-*Asteraceae* dicotyledonous plants, given *CmFtsH* genes exhibited closer evolutionary relationships with those of sunflower and lettuce.

**Table 2:** Numbers of *FtsH* genes in the four analyzed species genomes in total and eac h class.

Genome	Total Number	Subgroup							
		Class I	Class II	Class III	Class IV	Class V	Class VI	Class VII	Class VIII
*Chrysanthemum morifolium*	32	6	16	0	3	0	0	4	3
*Arabidopsis thaliana*	22	13	7	0	0	0	2	0	0
*Lactuca sativa*	13	4	2	0	0	0	0	2	5
*Helianthus annuus*	34	5	5	2	2	6	1	2	11

### 3.3 Analysis of the *FtsH* genes structure and motifs

The *FtsH* structures were comprehensively analyzed using the TBtools software to construct a map of *FtsH* structures (**Supplementary Figure 1**). Predictions made using MEME software revealed that the *CmFtsH* family contains 12 motifs that are highly conserved in terms of both type and sequence. With increasing homology, the similarity in gene motif arrangement also increased (**Supplementary Figure 1b**). The study identified nine *CmFtsH* genes (*CmFtsH-1*, *CmFtsH-2*, *CmFtsH-3*, *CmFtsH-4*, *CmFtsH-5*, *CmFtsH-7*, *CmFtsH-20*, *CmFtsH-21*, and *CmFtsH-22*) that exhibit an identical conserved motif composition and sequence, with all lacking motif 12. *CmFtsH-15*, *CmFtsH-16*, *CmFtsH-18*, *CmFtsH-19*, *CmFtsH-23*, and *CmFtsH-29* all lack motif 11 and motif 12. Both *CmFtsH-32* and *CmFtsH-17* retain only motif 6.

The *FtsH*_fam superfamily was identified within the structural domains of the genes *CmFtsH-1*, *CmFtsH-2*, *CmFtsH-3*, *CmFtsH-4*, *CmFtsH-5*, *CmFtsH-6*, *CmFtsH-7*, *CmFtsH-8*, *CmFtsH-9*, *CmFtsH-10*, and *CmFtsH-11* (**Supplementary Figure 1c**). In contrast, *CmFtsH-12* was found to be uniquely distinct, as it contained only the *FtsH*_superfamily structural domain. Meanwhile, *CmFtsH-13*, *CmFtsH-14*, *CmFtsH-15*, *CmFtsH-16*, and *CmFtsH-17* retained solely the core *FtsH* structural domain without additional superfamily features (**Supplementary Figure 1c**).

### 3.4 Chromosomal distribution of members of the *CmFtsH* genes

The study analyzed the distribution of *FtsH* gene family members within the chrysanthemum genome. The results revealed that the 32 *CmFtsH* genes were unevenly distributed across 16 out of the 26 chromosomes (**Supplementary Figure 2**). Chromosome 9 contained the largest number of *CmFtsH* genes, consisting of four members: *CmFtsH-22*, *CmFtsH-19*, *CmFtsH-27*, and *CmFtsH-28*. Chromosomes 12, 7, and 13 each contained three *CmFtsH* genes. These are *CmFtsH-12*, *CmFtsH-14*, and *CmFtsH-2* on chromosome 12. *CmFtsH-20*, *CmFtsH-18*, and *CmFtsH-26* are found on chromosome 7. *CmFtsH-32*, *CmFtsH-24*, and *CmFtsH-23* are located on chromosome 13. In contrast, chromosomes 2, 3, 14, 16, and 25 each contained a single *CmFtsH* gene.

### 3.5 Analysis of *Cis*-acting elements in the promoters of *CmFtsH* gene family members

The 2000 bp upstream regions of 32 *FtsH* genes, each with complete structural domains, were extracted from the chrysanthemum genome and analyzed for *cis*-acting elements using the PlantCARE database to better understand the chrysanthemum *FtsH* gene family’s regulatory mechanisms in response to abiotic stresses (**Supplementary Figure 3**). Twenty distinct functional elements were identified and classified into four major groups: Light-responsive elements constituted the majority, potentially linked to photosynthetic processes or photoperiod adaptation mechanisms; Hormone-responsive elements included responses to salicylic acid, methyl jasmonate (MeJA), gibberellins, among others, suggesting *FtsH* genes involvement in hormone-mediated developmental and stress responses; Abiotic stress response elements were related to defense mechanisms, drought tolerance, and cold acclimation, playing a vital role in regulating genes under adverse conditions like drought, low temperatures, and hypoxia; Other functional elements included those pertaining to meristem expression, seed-specific regulation, and MYB-binding sites, emphasizing the multifunctionality of the gene in developmental differentiation and metabolic regulation.

### 3.6 Synteny analysis of *CmFtsH* genes

Gene duplication is the primary mechanism for the expansion of gene families during genome evolution (Cannon et al., 2004). The study identified 33 pairs of collinear genes within the chrysanthemum genome (**Supplementary Figure 4**) and demonstrated substantial collinearity among *CmFtsH* genes distributed across different chromosomes of the same genome. These findings imply that chrysanthemum *FtsH* genes mainly consist of alleles or homozygous genes located on homologous chromosomes, highlighting the significance of gene amplification in evolutionary processes. For instance, *CmFtsH-12* on chromosome 12 is closely linked to *CmFtsH-1* on chromosome 11 and *CmFtsH-13* on chromosome 10. Similarly, *CmFtsH-19* on chromosome 9 is near *CmFtsH-17* on chromosome 8 and *CmFtsH-18* on chromosome 7. Moreover, *CmFtsH-27* found on chromosome 9 is connected to *CmFtsH-27* and *CmFtsH-17* on chromosome 8, as well as *CmFtsH-18* on chromosome 7. Additionally, *CmFtsH-27* is associated with *CmFtsH-9* on chromosome 25 and *CmFtsH-11* on chromosome 26 (**Supplementary Figure 4**). The findings suggest that the chrysanthemum *FtsH* genes probably originated from gene duplication, with the corresponding encoded proteins undergoing truncation events throughout evolutionary history.

### 3.7 GO annotation analysis of CmFtsH proteins

**Figure 2:**
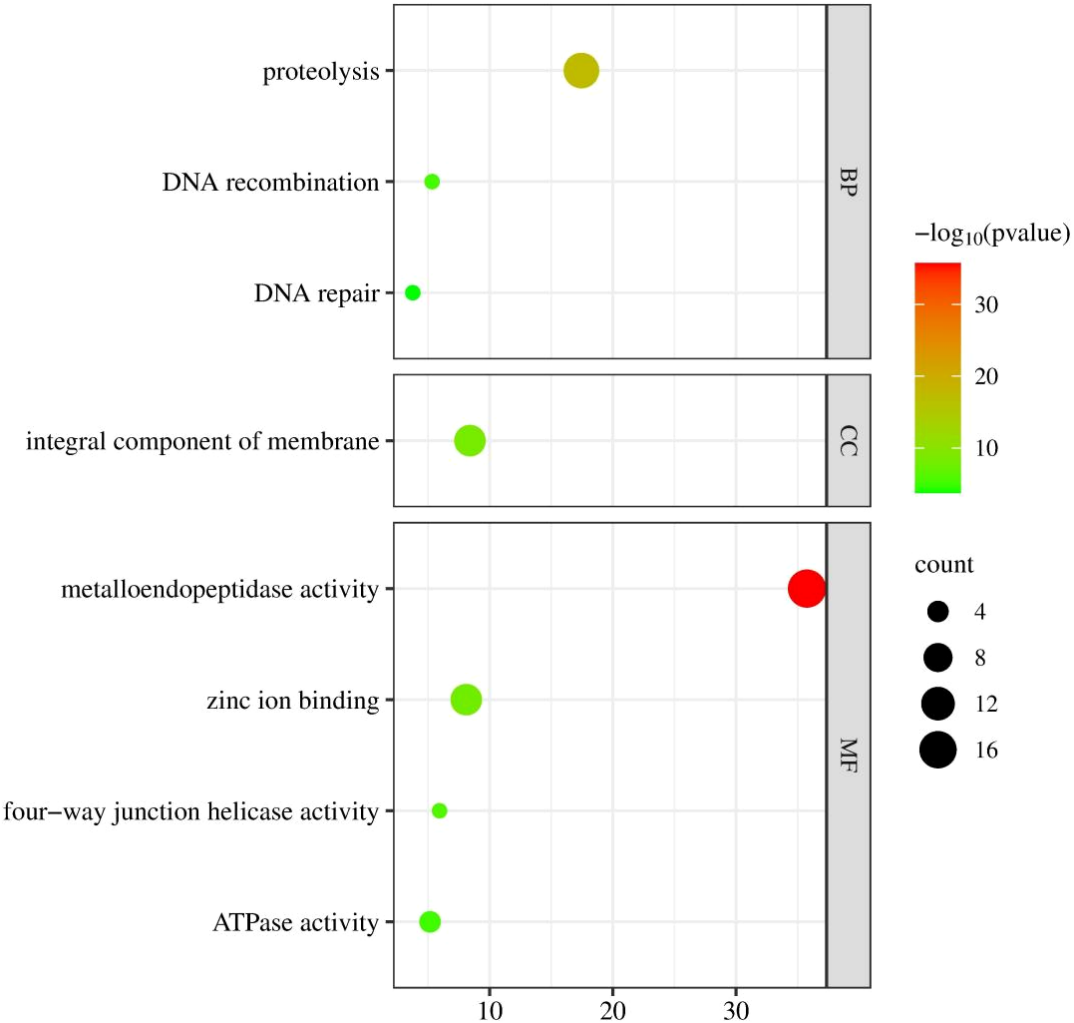
GO annotation analysis of 32 *FtsH* genes in chrysanthemum; MF represents molecular function, CC represents cellular component, and BP represents biological process.

The GO annotation study revealed that 32 CmFtsH proteins may be involved in various biological processes, cellular components, and molecular functions (**Figure 2**). Proteolysis is the main roles of CmFtsH proteins, according to an analysis of the biological processes these proteins mediate. Furthermore, CmFtsH proteins likely to be parts of the cell membrane, according to cellular localization studies. These proteins additionally perform out a variety of molecular functions, including as metallopeptidase activity, ATP-dependent peptidase activity, zinc ion binding ability, and tetrad complex forming enzyme activity.

### 3.8 Expression profiling of *CmFtsH* genes

**Figure 3:**
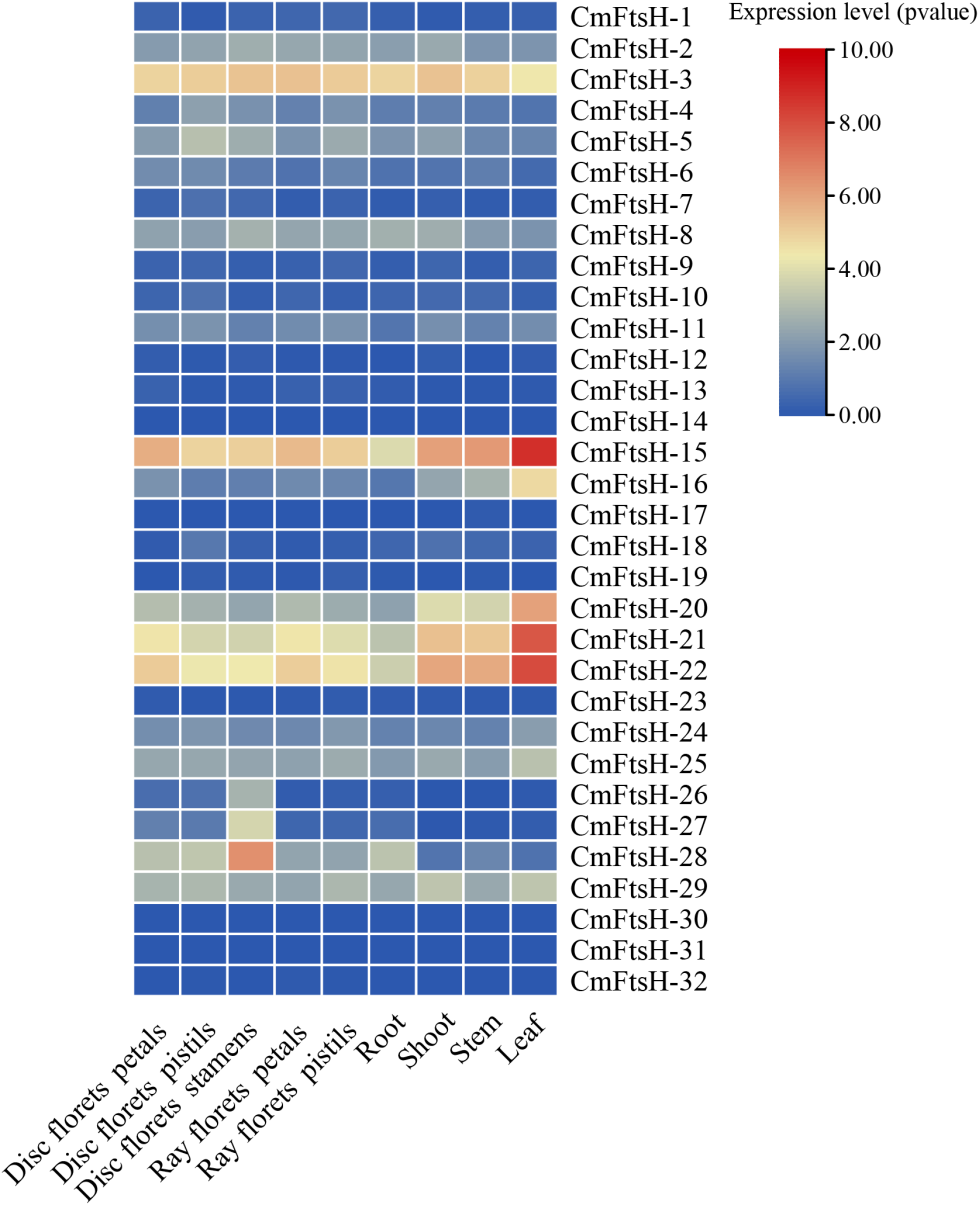
Expression profiling of the *CmFtsH* genes. The color gradient (red/yellow/blue) represented the gene expression level (from high to low).

A heatmap derived from the Chrysanthemum database transcriptome data (**Figure 3**) indicates that the majority of *CmFtsH* genes exhibit low expression levels or are unexpressed in the evaluated tissues, including tubular flower petals, tubular flower pistils, tubular flower stamens, ray flower petals, ray flower pistils, roots, stems, branches, and leaves. However, certain *CmFtsH* genes demonstrated elevated expression levels in specific tissues. For instance, *CmFtsH-15*, *CmFtsH-21*, and *CmFtsH-22* exhibited notable expression in leaves and additional tissues, while *CmFtsH-3* was detected in various tissues. The *CmFtsH-28* gene demonstrated elevated expression specifically in the pistils of the disc inflorescence. Conversely, *CmFtsH-14*, *CmFtsH-30*, and *CmFtsH-31* were undetected in the analyzed tissues. The findings suggest that distinct *CmFtsH* genes fulfill specific roles in chrysanthemum tissues, with certain genes displaying tissue-specific expression profiles.

### 3.9 Protein-protein interaction network of CmFtsH genes

To clarify the biological functions and regulatory networks of CmFtsH proteins, the protein-protein interactions of these proteins were systematically analyzed through complementary methodologies. The interaction network of CmFtsH proteins was predicted using Arabidopsis homologous FtsH proteins, as shown in **Supplementary Figure 5**. The analysis identified ten homologous CmFtsH proteins in Arabidopsis, corresponding to 52 proteins with functional interactions. The majority of CmFtsH proteins interact with multiple proteins. Notably, several interacting proteins, including HSP21, HSP70, RBL12, PHB1, PHB3, and PHB5, are associated with stress responses, facilitating plant adaptation under challenging conditions. Additionally, proteins such as TIC56, TIC20, TIC110, CLPP4, and CLPP1 play roles in chlorophyll synthesis.

### 3.10 *CmFtsH-15* positively regulates the cd resistance of chrysanthemum

*CmFtsH-15* was selected for comprehensive functional analysis due to its significant expression in leaf tissues and its suggested role in adapting to abiotic stress, as indicated by the characteristics of its promoter structure and linked protein interaction networks.

**Figure 4:**
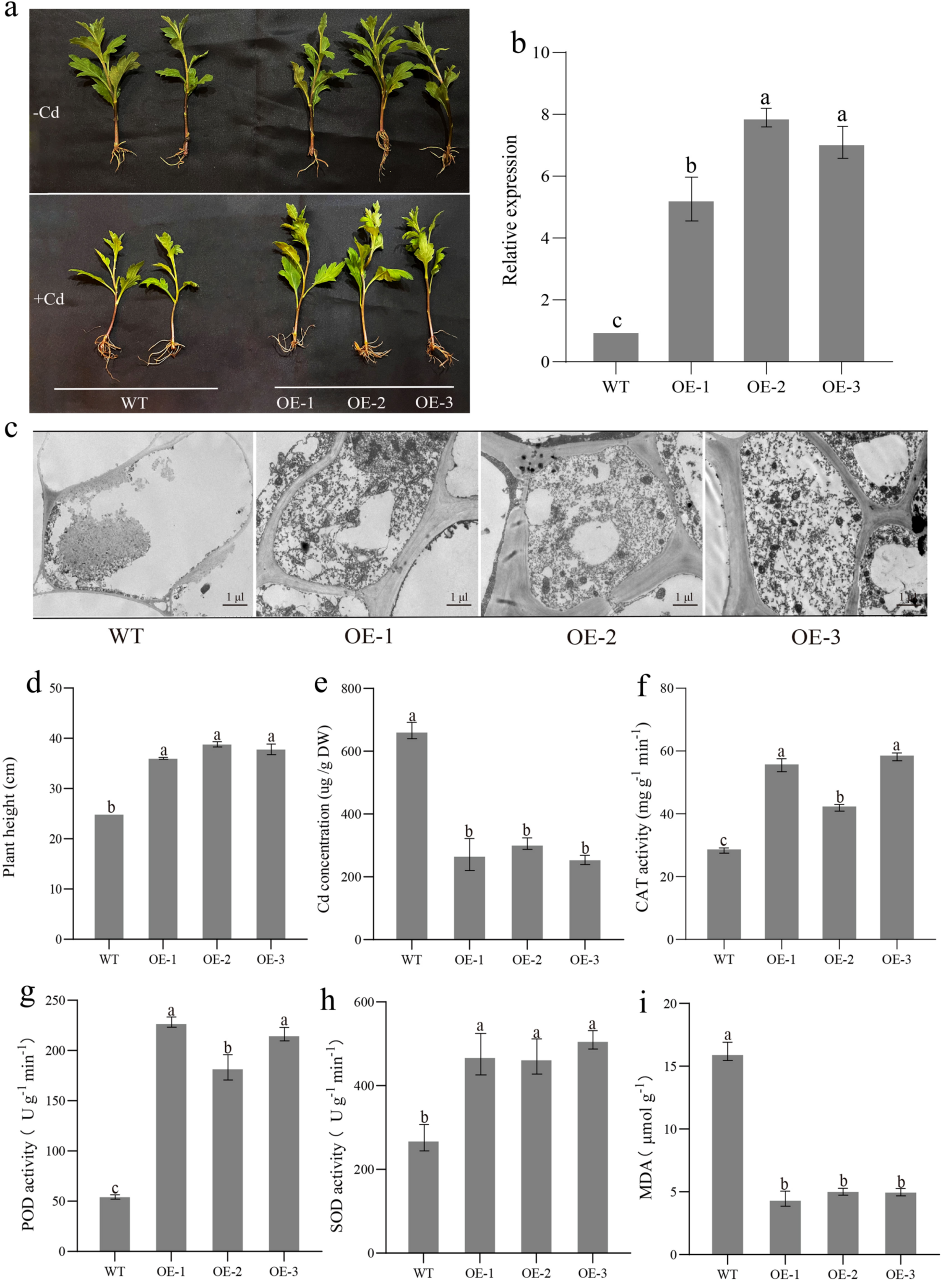
The phenotype of *CmFtsH-15* OE lines and WT under Cd stress. The *CmFtsH-15* OE lines and WT were transferred to a nutrient solution containing 2 mM Cd for 30 d. **(a)** Phenotypes of OE lines and WT; **(b)** Relative expression; **(c)** TEM were observed and representative images were chosen for analysis, Bars 1 μm; **(d)** Plant height; **(e)** Cd concentrations; **(f)** CAT activity; **(g)** POD activity; **(h)** SOD activity; **(i)** MDA content. Values are the mean ± SE (n=3); Letters indicate significant differences (p < 0.05).

This study constructed a *CmFtsH-15* overexpressing (OE) transgenic line. Phenotypic analysis revealed no significant differences in growth between the OE lines and the wild type (WT) under normal conditions. However, during Cd stress treatment, the WT exhibited notable growth inhibition, including reduced plant height and biomass, whereas the OE lines maintained better growth performance (**Figure 4a**). The expression of *CmFtsH-15* in the OE-1, OE-2, and OE-3 lines was 5.7, 8.1, and 6.3 fold higher, respectively, than in the WT (**Figure 4b**). In the WT cells, Cd stress caused damage such as mitochondrial swelling, disrupted chloroplast structures, and incomplete cell membranes. In contrast, the cells of the overexpression lines displayed relatively intact mitochondria, chloroplasts, and other organelle structures, with better-maintained cell membrane integrity (**Figure 4c**). This suggests that overexpression of *CmFtsH-15* gene enhanced cellular structural stability and mitigated Cd-induced cellular damage. The heights of OE-1, OE-2, and OE-3 lines increased by 46% to 51.2% compared to the WT (**Figure 4d**), while their Cd concentrations decreased by 55.1%, 57.2%, and 60.2%, respectively (**Figure 4e**). Moreover, the overexpression lines displayed significantly higher CAT, POD, and SOD activities than the WT, demonstrating an enhanced antioxidant enzyme system (**Figures 4f–h**). Conversely, the WT exhibited higher MDA content, indicative of greater lipid peroxidation, while the overexpression lines showed lower MDA levels (**Figure 4i**), underscoring the ability of *CmFtsH-15* overexpression to attenuate oxidative stress. Conclusively, the overexpression of the *CmFtsH-15* gene significantly improved chrysanthemum tolerance to Cd stress. The underlying mechanism likely involves mitigating Cd-induced oxidative damage by enhancing the plant’s antioxidant defense system while regulating Cd ion uptake and distribution. This effectively reduces Cd accumulation, preserves cell membrane integrity, and stabilizes key physiological and metabolic processes.

### 3.11 Verification of the interaction relationship between CmFtsH-15 and CmHSP70

**Figure 5:**
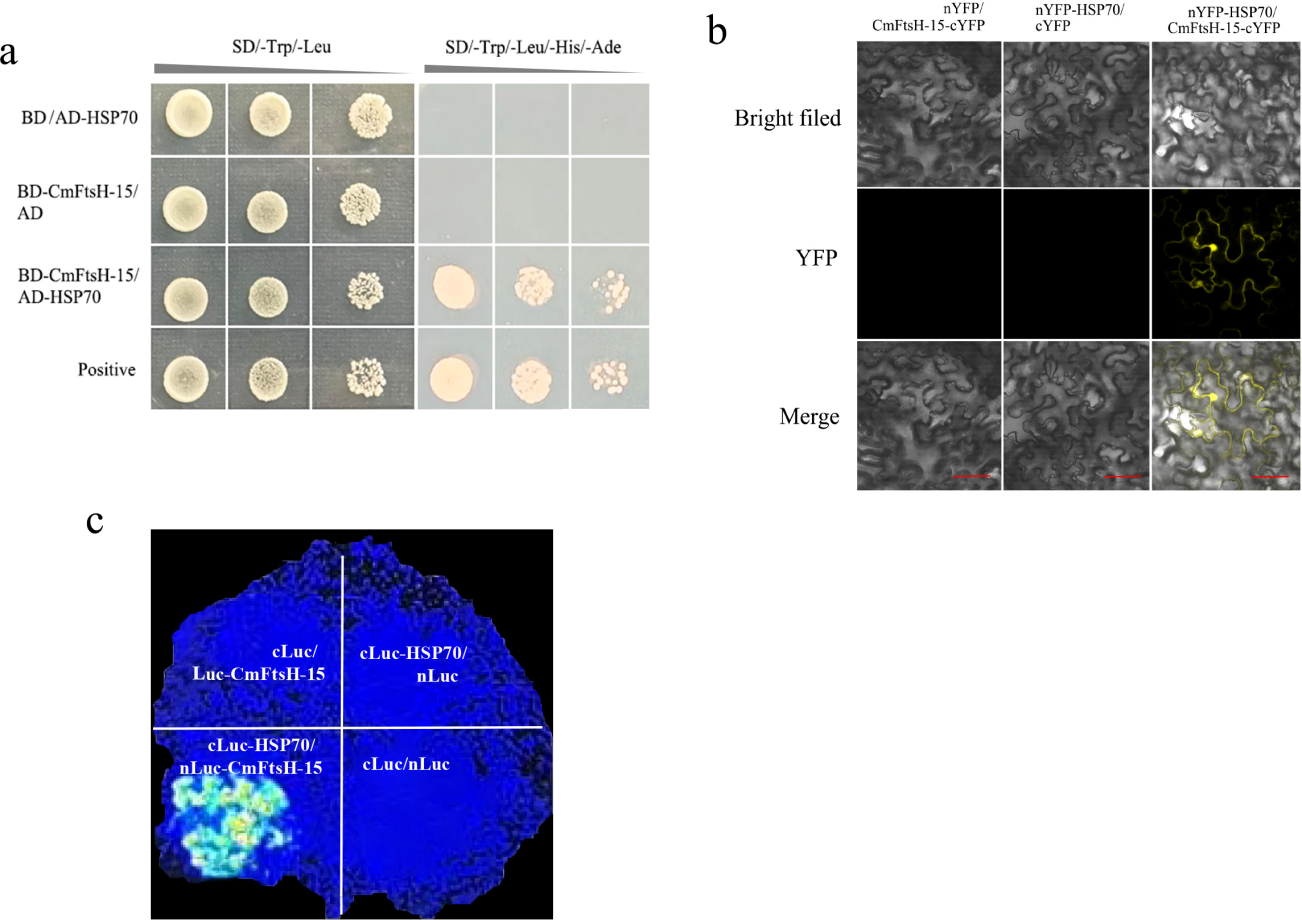
CmFtsH-15 interacts with CmHSP70. **(a)** Y2H showing the interaction of CmFtsH-15 and CmHSP70. Transformed yeast cells were grown on SD/-Trp/-Leu and SD/-Trp/-Leu/-His/-Ade media. **(b)** BiFC assays showing the interaction between CmFtsH-15 and CmHSP70. The nYFP-CmFtsH-15+pXY104 and pXY106+CmHSP70-cYFP were co-expressed in tobacco leaves. **(c)** Luc analysis of the interaction between CmFtsH-15 and CmHSP70 in tobacco leaves. The luminescent signal was collected at 48 h after infiltration.

The study confirmed the interaction between the CmFtsH-15 and CmHSP70 proteins through Y2H, Luc, and BiFC assays (**Figures 5a–c**). BiFC assays demonstrated that the interaction between CmFtsH-15 and CmHSP70 takes place at the cell membrane. Luc assays confirmed that strong luciferase activity was specifically observed in the nLuc-CmFtsH-15 and cLuc-CmHSP70 co-expression group, with no activity detected in the control groups (cLuc-CmHSP70/nLuc, cLuc/nLuc-CmFtsH-15, and nLuc/cLuc). Overall, these findings corroborate the interaction between CmFtsH-15 and CmHSP70.

## 4 Discussion

The FtsH protein family consists of ATP-dependent zinc metalloproteases that play a vital role in protein quality control, organelle maintenance, as well as stress response mechanisms in prokaryotic and eukaryotic organisms (Yi et al., 2022). These proteins are defined by their conserved AAA+ domains (ATPases Associated with Diverse Cellular Activities) and zinc-binding motifs, which facilitate the degradation of misfolded or damaged proteins, thereby preserving intracellular homeostasis (Langklotz et al., 2012; Janska et al., 2013). Within plants, FtsH proteins are integral to chloroplast development (Kato and Sakamoto, 2018), photosynthesis, and adaptation to abiotic stressors, including drought, elevated salinity, and heavy metal toxicity (Xiao et al., 2021; Chen et al., 2018).

Phylogenetic analysis indicates a substantial expansion of the *FtsH* gene family in chrysanthemum. Phylogenetic trees categorize FtsH proteins from *Chrysanthemum morifolium*, *Arabidopsis thaliana*, *Lactuca sativa*, and *Helianthus annuus* into eight distinct subfamilies; approximately 50% of Chrysanthemum members are localized in the second subfamily, suggesting a lineage-specific expansion within *Asteraceae* (**Figure 1**) (Pu et al., 2022). Members of the *CmFtsH* family range in molecular weight from 18.97 to 99.03 kDa. Though exhibiting significant structural diversity, most contain conserved motifs and the *FtsH*_fam_superfamily domain, indicating preservation of their fundamental proteolytic function (**Table 1**, **Supplementary Figure 1**) (Pu et al., 2022; Li et al., 2025). Dilations in gene duplication events significantly contributed to the expansion of this family, with 33 pairs of collinear gene pairs documented (**Supplementary Figure 4**) (Song et al., 2023). The promoter regions are abundant in light, hormone, and abiotic stress response elements (**Supplementary Figure 3**). Based on the established role of FtsH proteins in stress-induced degradation of damaged proteins (Huang et al., 2024; Krynická and Komenda, 2024), it is hypothesized that *CmFtsH* functions as a regulator in the response of chrysanthemums to abiotic stress.

Abiotic stresses, including heavy metal toxicity, severely disrupt plant cellular homeostasis and induce oxidative damage, impairing metabolic functions, thus inhibiting plant growth and development, and reducing productivity (Gill et al., 2012; Haider et al., 2021). Among these, Cd, as a non-essential heavy metal element, exhibits significant toxicity to plants attributed to its high environmental mobility and persistence (Jia et al., 2020; Mushtaq et al., 2025). This study provides a functional characterization of the *CmFtsH-15* gene in chrysanthemum. Physiological evidence indicates that overexpression of *CmFtsH-15* significantly reduced Cd accumulation in chrysanthemums, decreasing levels by 55.1%–60.2%. while enhancing the activities of CAT, POD, and SOD, and reducing MDA content under Cd stress (**Figures 4e–i**). TEM further revealed that under Cd stress, the cellular structural integrity of the overexpression system was effectively maintained, contrasted with wild-type cells exhibiting significant organelle swelling and membrane disintegration (**Figure 4c**). The findings indicate that *CmFtsH-15* enhances chrysanthemum’s Cd tolerance through a dual mechanism: reducing Cd accumulation within plants while simultaneously boosting antioxidant enzyme activity. This action effectively suppresses reactive oxygen species accumulation and membrane lipid peroxidation, thereby maintaining cellular structural stability. This functional mechanism shows parallels with other recognized heavy metal response genes, such as metallothioneins and plant chelate peptidases (Liu et al., 2016; Wu et al., 2024). However, as a member of the protease family, the mechanism of action of *CmFtsH-15* may be more focused on maintaining protein homeostasis. Consistent with research on FtsH proteases in other species, these proteins maintain proteome balance by degrading oxidatively damaged proteins (Lindahl et al., 2000; Malnoë et al., 2014). Therefore, *CmFtsH-15* probably enhances chrysanthemum tolerance to Cd stress by clearing Cd-induced oxidatively damaged proteins and maintaining intracellular protein homeostasis. However, the present investigation only demonstrates that *CmFtsH-15* improves plant Cd tolerance through overexpression experiments, with no comparable loss-of-function analyses such as gene silencing or deletion. As a result, it is difficult to assess the functional significance of this gene in the Cd stress response and its likely genetic processes. Future research using RNA interference or CRISPR/Cas9 gene editing technologies will explore more deeply into the role of *CmFtsH-15* in Cd stress adaptation.

This study further explored the mechanism underlying CmFtsH-15, revealing a direct interaction with CmHSP70 (**Figure 5**). As a core component of the protein chaperone system, HSP70 plays an essential role in protein folding, repair, and degradation (Usman et al., 2017). This interaction uncovers a synergistic protein quality control network: under Cd stress, HSP70 recognizes and binds misfolded proteins to attempt repair, and irreparable proteins are degraded via FtsH-mediated pathways, thus effectively alleviating protein toxicity. This mechanism is highly conserved evolutionarily, similar to the model in *Escherichia coli*, where FtsH and DnaK (an HSP70 homolog) cooperatively clear abnormal proteins (Rodriguez et al., 2008). The HSP70 family has also been extensively reported in plant heavy metal stress responses, which enhance tolerance by stabilizing target proteins (Yin et al., 2023; Cho and Choi, 2009). Consequently, the discovery of the CmFtsH-15-CmHSP70 interaction module unveils a conserved strategy for protein homeostasis in plants.

## 5 Conclusion

A comprehensive analysis of the *FtsH* gene family in chrysanthemum identified 32 *CmFtsH* genes, highlighting their structural diversity and phylogenetic relationships. These genes exhibit closer relationships with other *Asteraceae* species than with non-*Asteraceae* plants, suggesting lineage-specific expansions that enhance adaptability. The genes are unevenly distributed across 16 of the 26 chromosomes and exhibit significant variations in intron number and gene sequence length. Promoter analysis identified multiple *cis*-acting elements, suggesting their potential roles in stress responses. Expression profiling demonstrated tissue-specific and stress-responsive expression patterns, with certain genes exhibiting high expression in specific tissues and others responding to Cd stress. Functional validation of *CmFtsH-15* confirmed its role in enhancing Cd tolerance, achieved through increased antioxidant activity and reduced Cd accumulation in transgenic plants. Furthermore, the interaction between CmFtsH-15 and CmHSP70 indicates a cell protection mechanism that enhances tolerance to Cd through effective quality control of proteins and maintenance of organelles. These findings provide a foundation for further research into the *FtsH* gene family and its applications in improving stress tolerance in chrysanthemum and related species. Future studies will further validate the functional necessity of *CmFtsH-15* through gene silencing and knockout approaches, and elucidate the molecular mechanisms underlying its role in mediating Cd stress tolerance.

## Data Availability

The raw data supporting the conclusions of this article will be made available by the authors, without undue reservation.
