# Supplementary material for: Identification of the FtsH gene family in chrysanthemums and functional analysis of CmFtsH-15 under cadmium stress
Source: Front Plant Sci. 2026 Mar 13;17:1768126. doi: 10.3389/fpls.2026.1768126 (PMC13021575; doi:10.3389/fpls.2026.1768126)
Supplement: Supplementary file 1 [file Table1.doc]

# **Table S1 Primer sets used in this study.**

| **Name** | **Sequence (5’-3’)*** | **Application** |
| --- | --- | --- |
| OE-CmFtsH-15-F | GAGATCTATGAAATTTTTTCAGCAG | Overexpression of *CmFtsH-15* |
| OE-CmFtsH-15-R | CGCTCGAGTCATACACTTACTGGACTTGG |
| CmEF1α-F | TTTTGGTATCTGGTCCTGGAG | qPCR of Internal reference gene |
| CmEF1α-R | CCATTCAAGCGACAGACTCA |
| qCmFtsH-15-F | TGGGAAACCGTGTTCAGAGA | qPCR of *CmFtsH-15* |
| qCmFtsH-15-R | GCATTGTGTGCTGCAAAGTC |
| pGBKT7-CmFtsH-15-F | CATGGAGGCCGAATTCATGAAATTTTTTCAGCAG | Y2H assay |
| pGBKT7-CmFtsH-15-R | GCAGGTCGACGGATCCTCATACACTTACTGGACTTGG |
| pGADT7-CmHSP70-F | GGAGGCCAGTGAATTCATGGTTAACCACTTTGTTCAA |
| pGADT7-CmHSP70-R | CGAGCTCGATGGATCCTCAAGACCAAGAGACAATGGG |
| BiFC-CmFtsH-15-F | ATCGAGGACGCCGGCGGATCCTCATACACTTACTGG | BiFC assay |
| BiFC-CmFtsH-15-R | CAGGTCGACTCTAGAGGATCCTCATACACTTACTGGACTTGG |
| BiFC-CmHSP70-F | ATTACAGGTACCCGGGGATCCATGGTTAACCACTTTGTTCAA |
| BiFC-CmHSP70-R | CAGGTCGACTCTAGAGGATCCTCAAGACCAAGAGACAATGGG |
| Luc-CmFtsH-15-F | CACGGGGGACGAGCTCGGTACCATGAAATTTTTTCAGCAG | Luc assay |
| Luc-CmFtsH-15-R | ACGAAAGCTCTGCAGGTCGACTCATACACTTACTGGACTTGG |
| Luc-CmHSP70-F | TACGCGTCCCGGGGCGGTACCATGGTTAACCACTTTGTTCAA |
| Luc-CmHSP70-R | ACGAAAGCTCTGCAGGTCGACAGACCAAGAGACAATGGG |


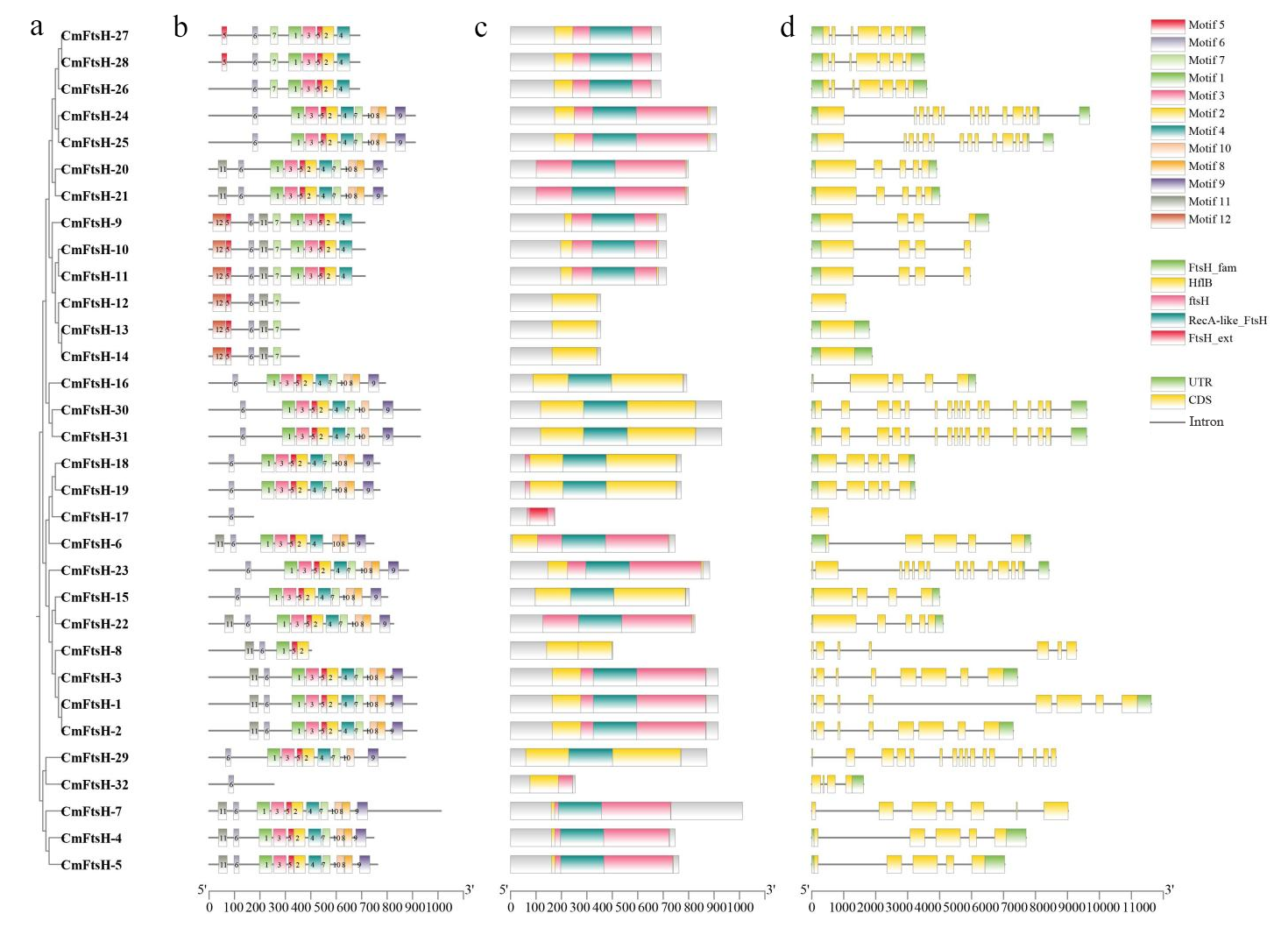


**Figure S1.** Phylogenetic relationships, conserved motifs, conserved sequences and gene structure of the *CmFtsHs*. (a) Construction of a rootless neighbour-joining phylogenetic tree comprising 32 *CmFtsH* gene sequences. (b) Distribution of conserved motifs within the *CmFtsH* gene sequences. The differently coloured boxes represent different bases, and the motif numbers of the genes are shown in the coloured boxes. (c) Conserved domain of *CmFtsHs*; The differently coloured boxes represent different conserved sequences. (d) Exon/ Intron structures of *CmFtsHs*. The green boxes represent exons, and the same-length black lines represent introns. The lengths of the exons can be inferred from the scale at the bottom.


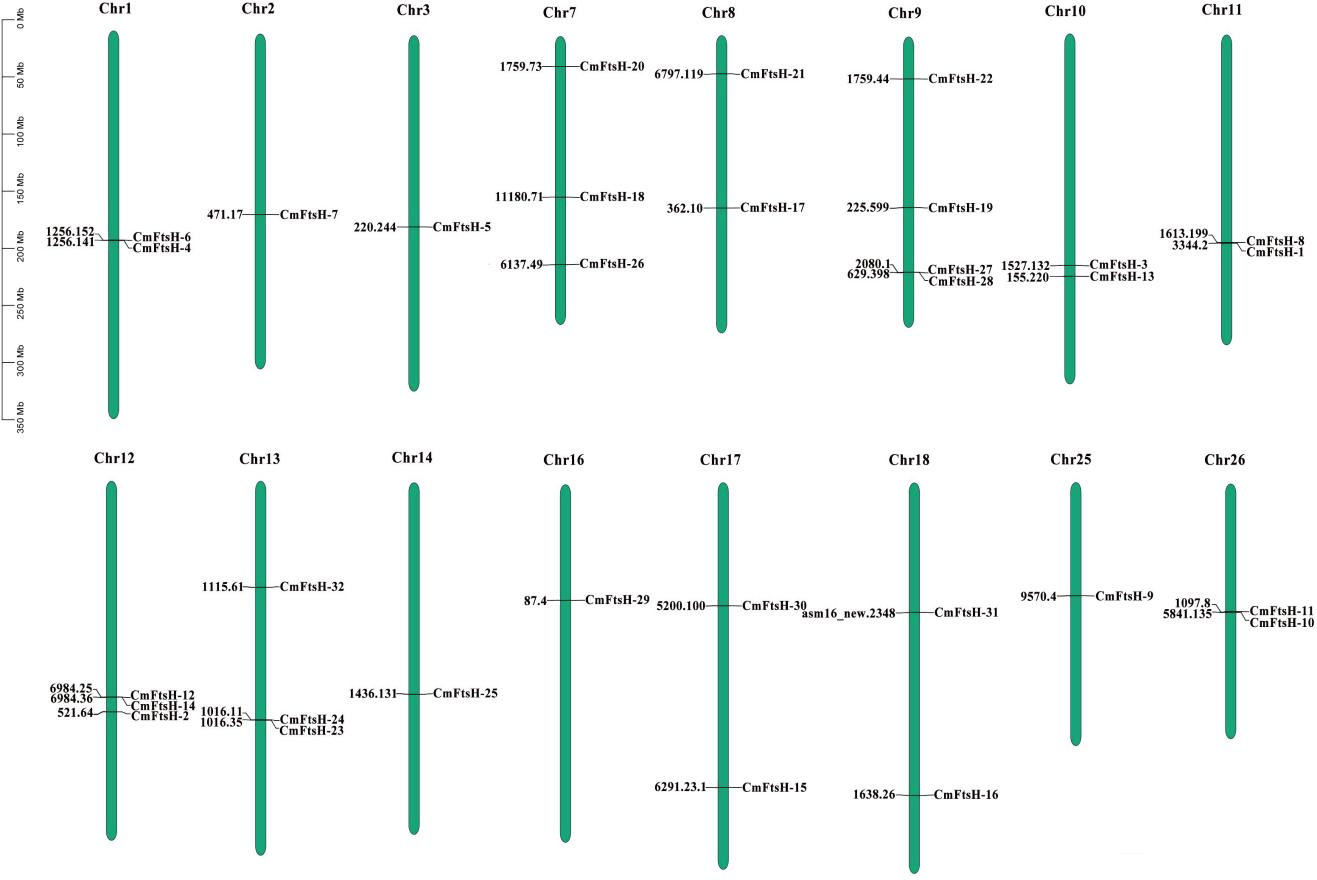


**Figure S2.** Genomic distribution of *CmFtsHs* across the sixteen chrysanthemum chromosomes. The list of organisms by chromosome count is shown on the upper-left side of each chromosome. The chromosomal locations of the *CmFtsHs* were determined according to the physical location of each gene.


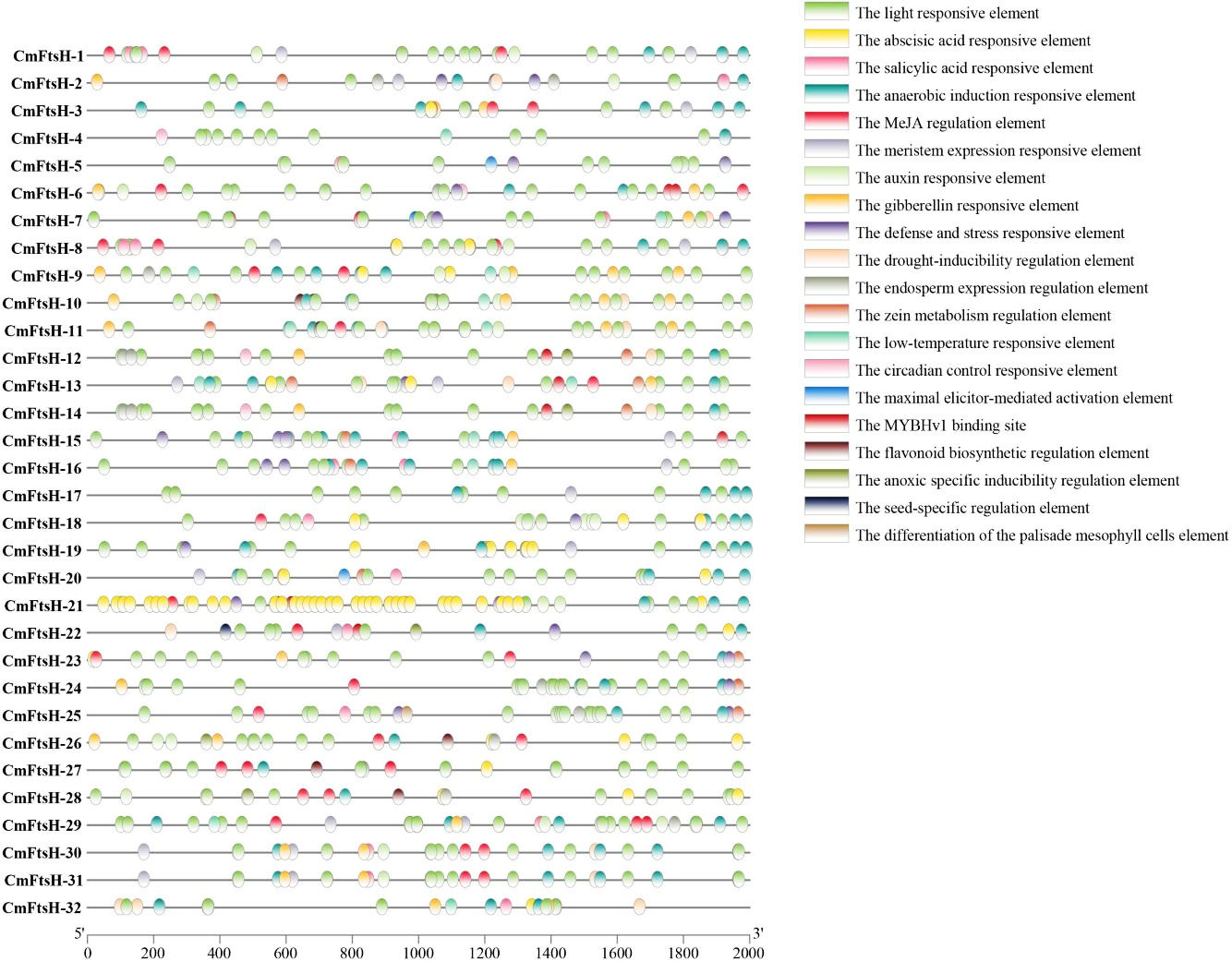


**Figure S3.** Promoter *cis*-acting elements analysis of the chrysanthemum *FtsHs*. The different coloured boxes indicated different types of *cis*-acting elements.


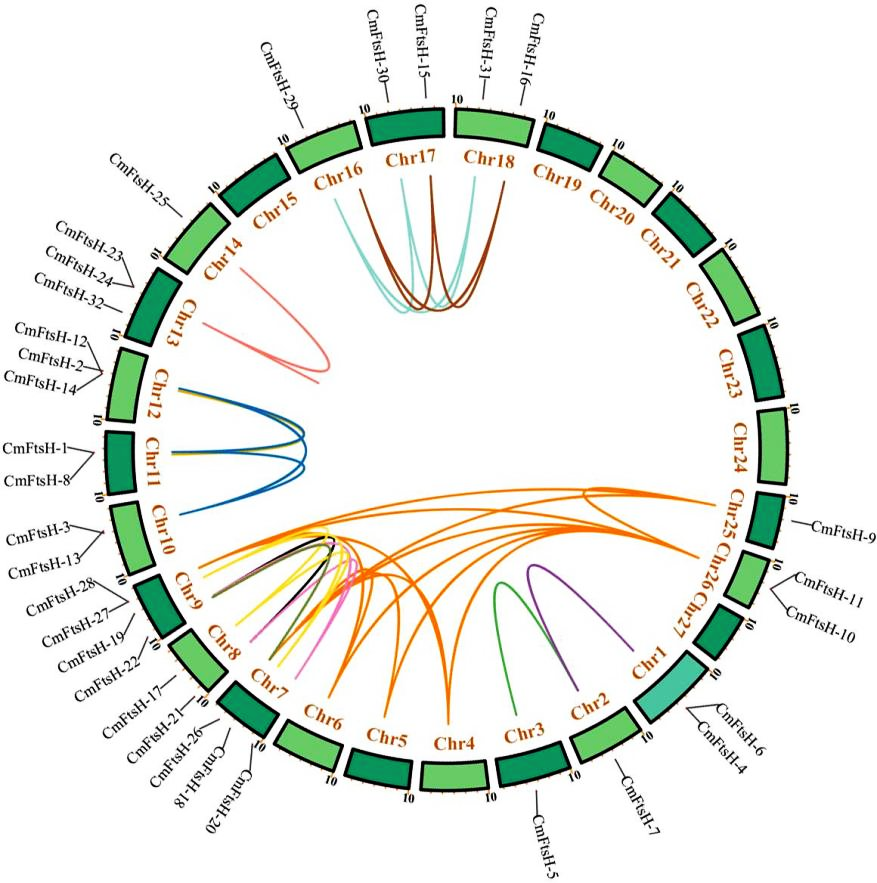


**Figure S4.** Location, homologous gene pairs of *FtsHs* in chrysanthemum genomes. All *CmFtsHs* were mapped to their respective location in the chrysanthemum genomes. Homologous genes were linked with corresponding colors. The graph was generated via Circos.


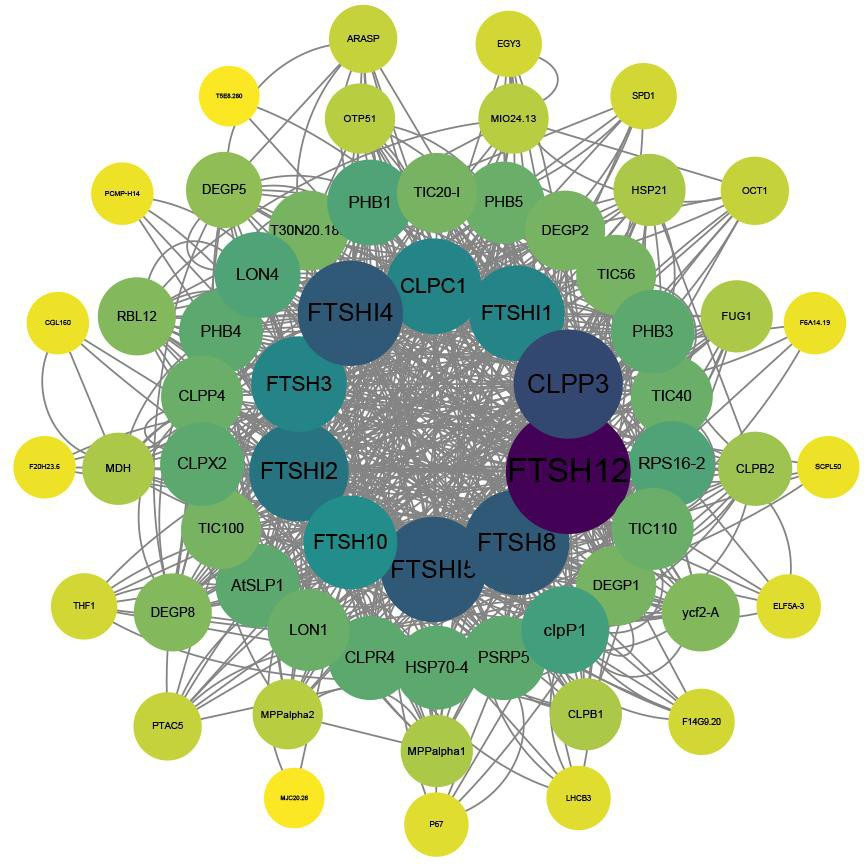


**Figure S5.** Protein-protein interaction network of CmFtsHs. The different coloured boxes indicated different types of protein.
